# Hemosuccus pancreaticus treated using EUS-guided coil embolization and glue injection

**DOI:** 10.1016/j.igie.2024.02.008

**Published:** 2024-02-17

**Authors:** Harishankar Gopakumar, Hasan Shoaib, Srinivas R. Puli

**Affiliations:** Department of Gastroenterology and Hepatology, University of Illinois College of Medicine at Peoria, Peoria, Illinois, USA

A 63-year-old man with recurrent alcohol-induced pancreatitis presented with complaints of epigastric abdominal pain and melena. His hemoglobin level was 10.20 g/dL. A CT of the abdomen showed a pancreatic pseudoaneurysm measuring 2.6 × 2.4 cm at the uncinate process (Fig. 1), communicating with a branch of the inferior pancreaticoduodenal arcade (Fig. 2). This pseudoaneurysm also resulted in the dilation of the pancreatic duct (PD) to 5 mm because of mass effect.

**Figure 1:**
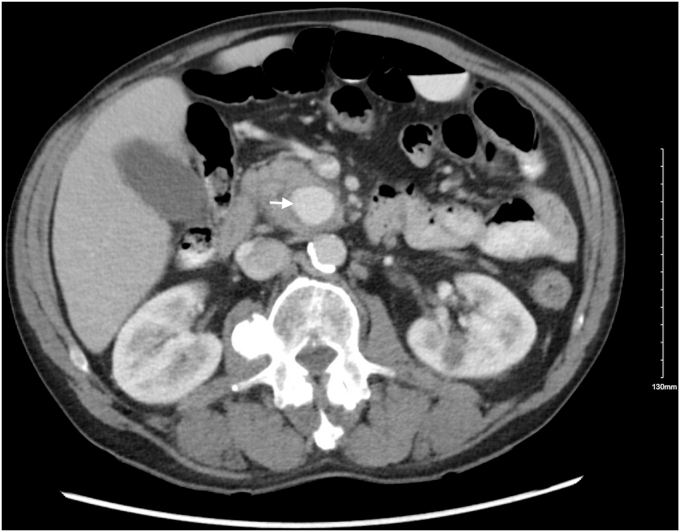
CT of the abdomen showing a pseudoaneurysm measuring 2.6 × 2.4 cm in the uncinate process of the pancreas.

**Figure 2:**
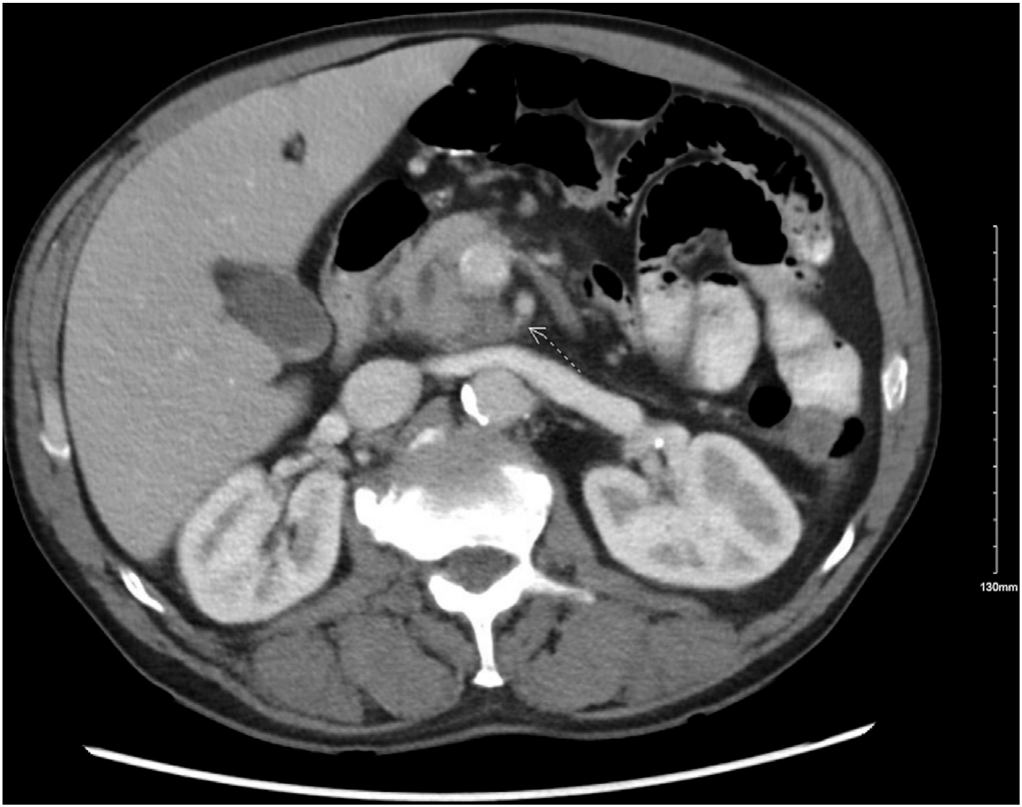
Pseudoaneurysm communicating with a branch of the inferior pancreaticoduodenal arcade.

EGD showed active extravasation of blood from the major duodenal papilla (Fig. 3), indicating a fistula between the PD and the pseudoaneurysm. After a multidisciplinary review with interventional radiology, we decided to proceed with an endoscopy-directed therapeutic approach because the pseudoaneurysm involved a small branch of the inferior pancreaticoduodenal arcade, making an angiographic approach by interventional radiology challenging.

**Figure 3:**
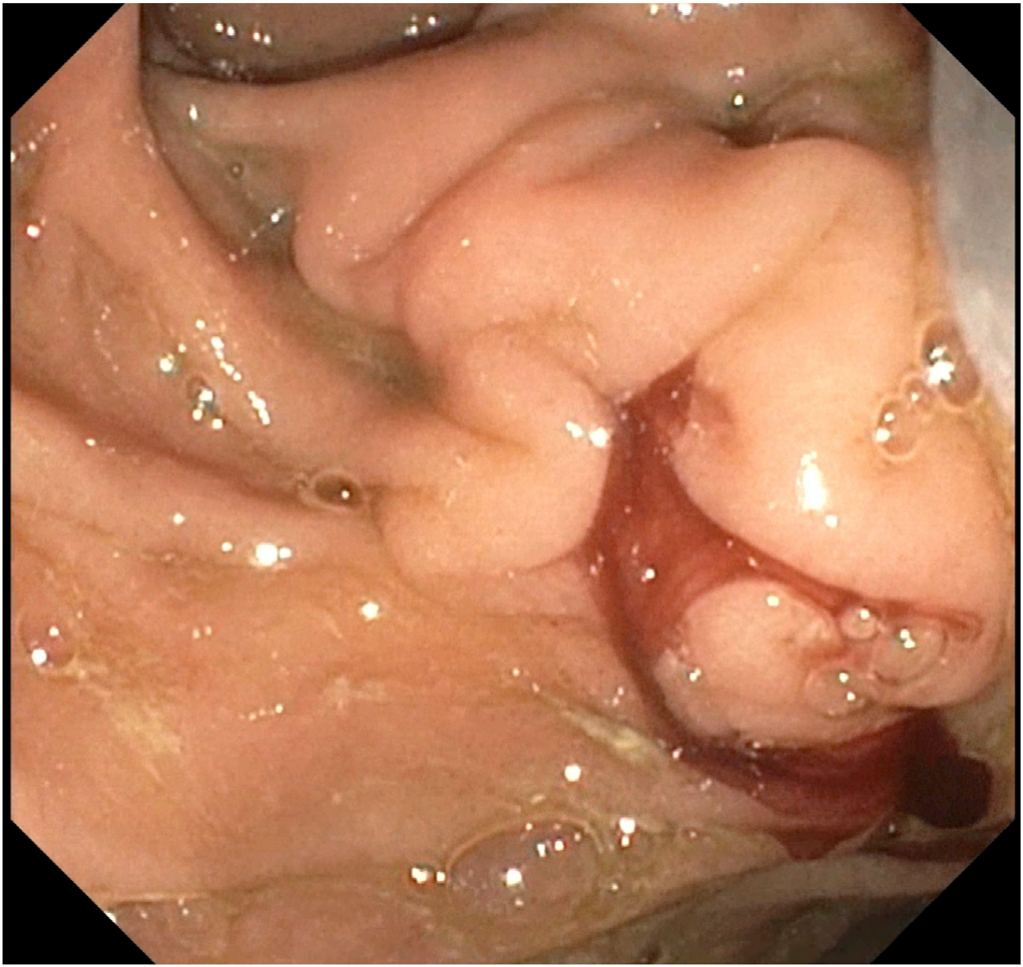
EGD showed active extravasation of blood from the major duodenal papilla.

The patient underwent ERCP with sphincterotomy and placement of PD and common bile duct stents. A common bile duct stent was used to assist in PD cannulation and to reduce the risk of distal migration of the PD stent. EUS showed a 2.5 × 2.5 cm pseudoaneurysm in the head of the pancreas, corresponding to the findings on abdominal CT (Fig. 4).

**Figure 4:**
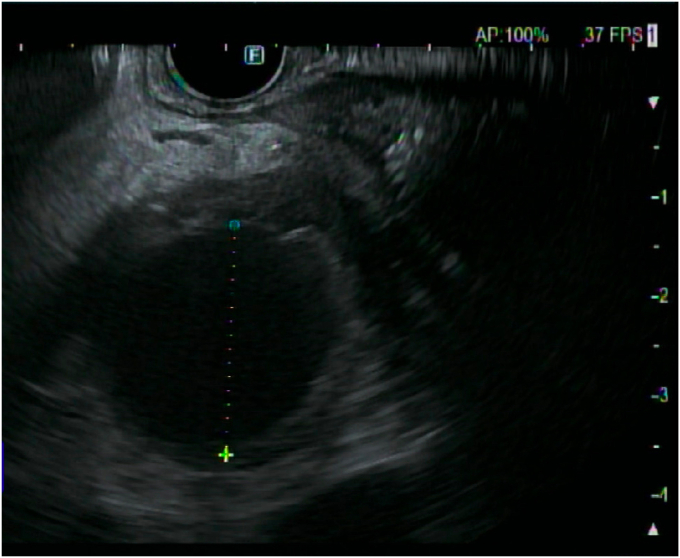
EUS showed a 2.5 × 2.5 cm pseudoaneurysm in the head of the pancreas.

EUS-guided coiling followed by glue injection into the pseudoaneurysm resulted in prompt resolution of the bleeding (Figs. 5 and 6). A standard 19-gauge EUS needle was used for this procedure. Two embolization coils, the Nester Embolization Coil (16 mm × 14 cm; Cook Medical, Bloomington, Ind, USA) and the Mreye Embolization Coil (20 mm × 20 cm; Cook Medical), were introduced into the pseudoaneurysm, followed by .3 mL histoacryl glue injection. Because no guidelines or robust data exist from randomized control trials to guide this procedure, the decision regarding the number of coils was based on the endoscopist’s experience. After deploying 2 coils, histoacryl was injected into the pseudoaneurysm to further reduce the blood flow visualized in real-time on EUS. The procedure was determined to be adequate after observing an absence of blood flow and increasing hyperechoic appearance in the region of the pseudoaneurysm on EUS, indicative of significantly diminished or absent blood flow into the pseudoaneurysm (Video 1, available online at www.igiejournal.org). At the 1-month follow-up, the patient remained asymptomatic with improved hemoglobin of 13 g/dL and no evidence of GI blood loss.

**Figure 5:**
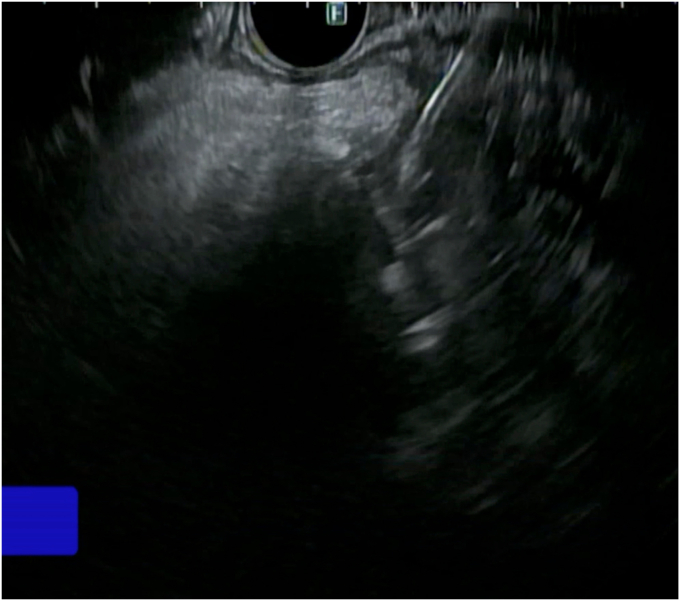
EUS images showing coils deployed in the pseudoaneurysm.

**Figure 6:**
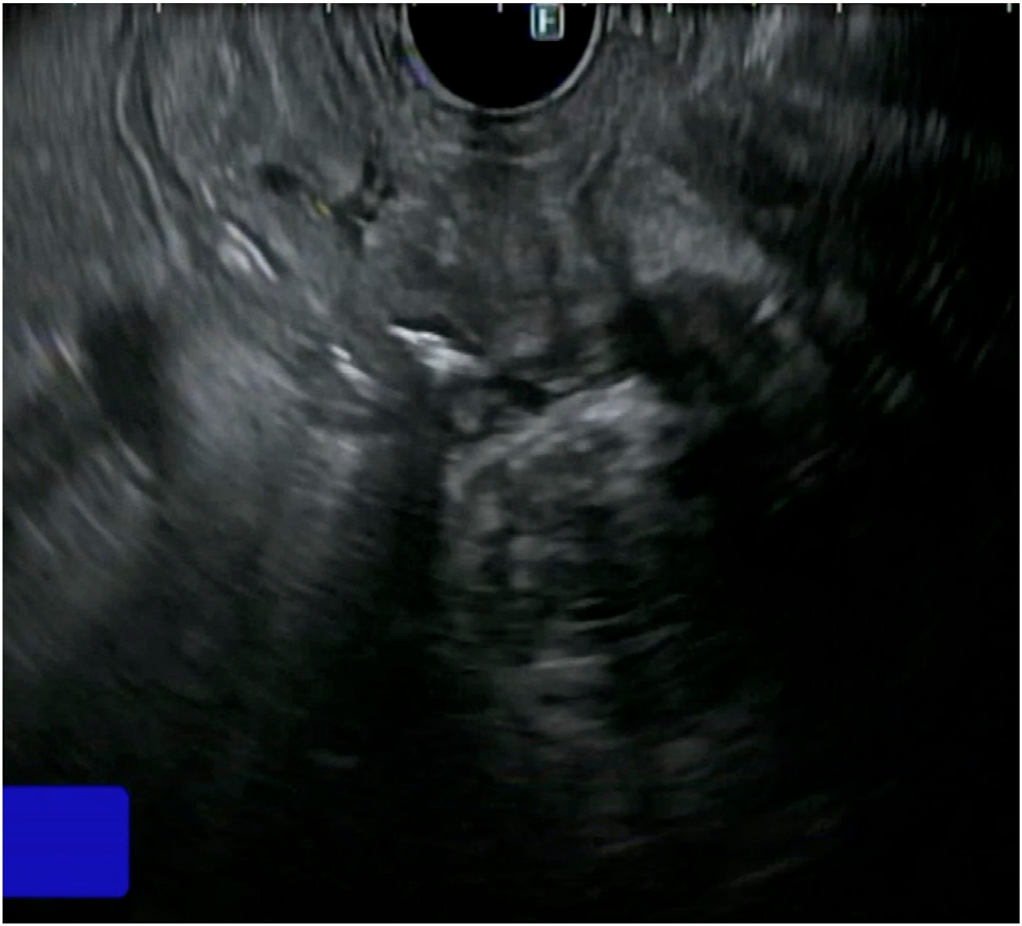
EUS images showing coils and histoacryl glue in the pseudoaneurysm.

## Disclosure

All authors disclosed no financial relationships.

## Supplementary data

Video 1 Video demonstrating successful EUS-guided coil embolization and glue injection of pancreatic pseudoaneurysm.

